# Supplementary material for: Partial Reduction in BRCA1 Gene Dose Modulates DNA Replication Stress Level and Thereby Contributes to Sensitivity or Resistance
Source: Int J Mol Sci. 2022 Nov 1;23(21):13363. doi: 10.3390/ijms232113363 (PMC9656774; doi:10.3390/ijms232113363)
Supplement: Supplementary file 1 [file ijms-23-13363-s001.zip › ijms-1982598-supplementary.pptx]

## Slide 1
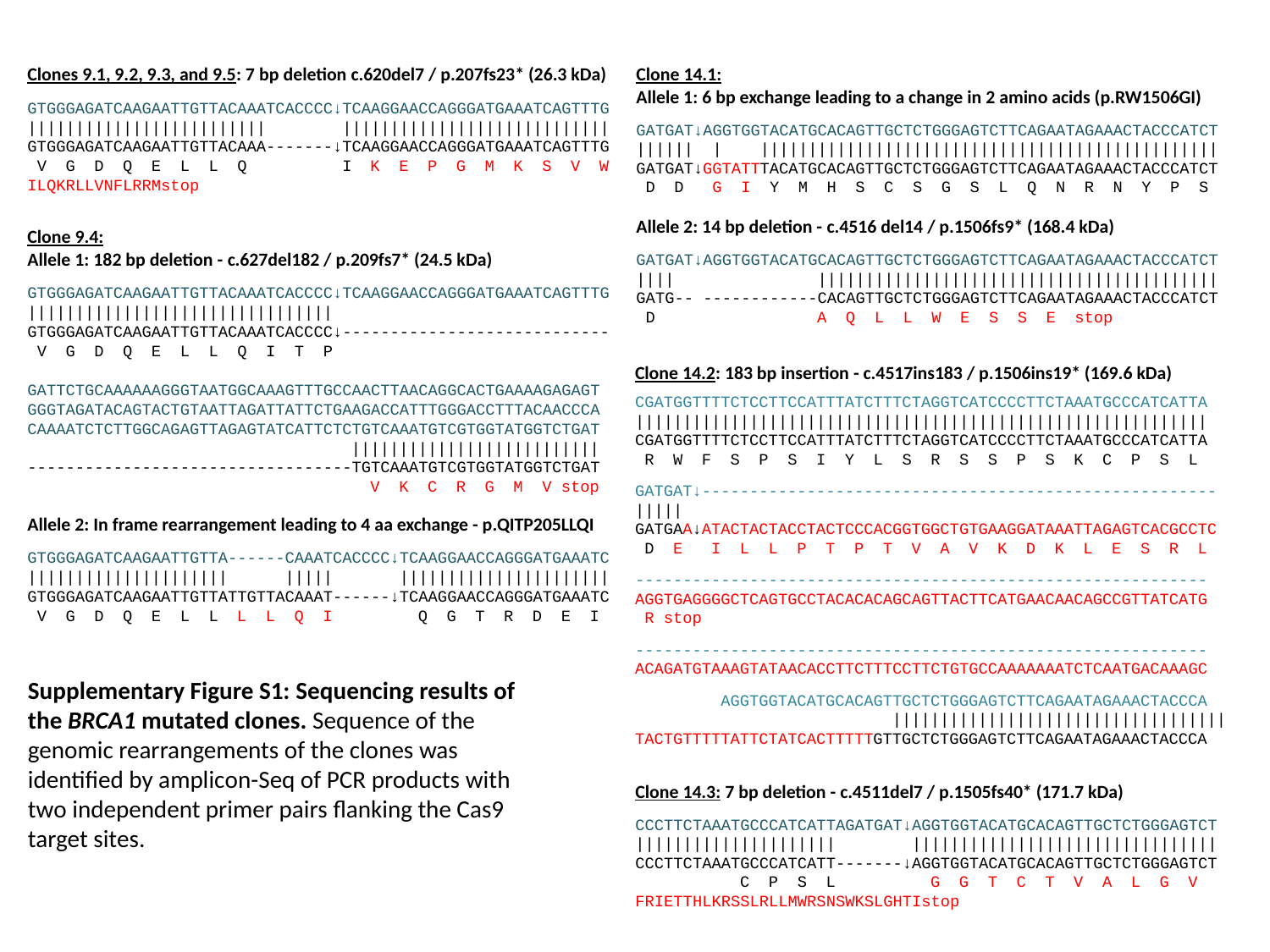

Clones 9.1, 9.2, 9.3, and 9.5: 7 bp deletion c.620del7 / p.207fs23* (26.3 kDa)
GTGGGAGATCAAGAATTGTTACAAATCACCCC↓TCAAGGAACCAGGGATGAAATCAGTTTG
||||||||||||||||||||||||| ||||||||||||||||||||||||||||
GTGGGAGATCAAGAATTGTTACAAA-------↓TCAAGGAACCAGGGATGAAATCAGTTTG
 V G D Q E L L Q I K E P G M K S V W
ILQKRLLVNFLRRMstop
Clone 14.1:
Allele 1: 6 bp exchange leading to a change in 2 amino acids (p.RW1506GI)
GATGAT↓AGGTGGTACATGCACAGTTGCTCTGGGAGTCTTCAGAATAGAAACTACCCATCT
|||||| | ||||||||||||||||||||||||||||||||||||||||||||||||
GATGAT↓GGTATTTACATGCACAGTTGCTCTGGGAGTCTTCAGAATAGAAACTACCCATCT
 D D G I Y M H S C S G S L Q N R N Y P S
Allele 2: 14 bp deletion - c.4516 del14 / p.1506fs9* (168.4 kDa)
GATGAT↓AGGTGGTACATGCACAGTTGCTCTGGGAGTCTTCAGAATAGAAACTACCCATCT
|||| ||||||||||||||||||||||||||||||||||||||||||
GATG-- ------------CACAGTTGCTCTGGGAGTCTTCAGAATAGAAACTACCCATCT
 D A Q L L W E S S E stop
Clone 9.4:
Allele 1: 182 bp deletion - c.627del182 / p.209fs7* (24.5 kDa)
GTGGGAGATCAAGAATTGTTACAAATCACCCC↓TCAAGGAACCAGGGATGAAATCAGTTTG
||||||||||||||||||||||||||||||||
GTGGGAGATCAAGAATTGTTACAAATCACCCC↓----------------------------
 V G D Q E L L Q I T P
GATTCTGCAAAAAAGGGTAATGGCAAAGTTTGCCAACTTAACAGGCACTGAAAAGAGAGT
GGGTAGATACAGTACTGTAATTAGATTATTCTGAAGACCATTTGGGACCTTTACAACCCA
CAAAATCTCTTGGCAGAGTTAGAGTATCATTCTCTGTCAAATGTCGTGGTATGGTCTGAT
 ||||||||||||||||||||||||||
----------------------------------TGTCAAATGTCGTGGTATGGTCTGAT
 V K C R G M V stop
Allele 2: In frame rearrangement leading to 4 aa exchange - p.QITP205LLQI
GTGGGAGATCAAGAATTGTTA------CAAATCACCCC↓TCAAGGAACCAGGGATGAAATC
||||||||||||||||||||| ||||| ||||||||||||||||||||||
GTGGGAGATCAAGAATTGTTATTGTTACAAAT------↓TCAAGGAACCAGGGATGAAATC
 V G D Q E L L L L Q I Q G T R D E I
Clone 14.2: 183 bp insertion - c.4517ins183 / p.1506ins19* (169.6 kDa)
CGATGGTTTTCTCCTTCCATTTATCTTTCTAGGTCATCCCCTTCTAAATGCCCATCATTA
||||||||||||||||||||||||||||||||||||||||||||||||||||||||||||
CGATGGTTTTCTCCTTCCATTTATCTTTCTAGGTCATCCCCTTCTAAATGCCCATCATTA
 R W F S P S I Y L S R S S P S K C P S L
GATGAT↓------------------------------------------------------
|||||
GATGAA↓ATACTACTACCTACTCCCACGGTGGCTGTGAAGGATAAATTAGAGTCACGCCTC
 D E I L L P T P T V A V K D K L E S R L
------------------------------------------------------------
AGGTGAGGGGCTCAGTGCCTACACACAGCAGTTACTTCATGAACAACAGCCGTTATCATG
 R stop
------------------------------------------------------------
ACAGATGTAAAGTATAACACCTTCTTTCCTTCTGTGCCAAAAAAATCTCAATGACAAAGC
 AGGTGGTACATGCACAGTTGCTCTGGGAGTCTTCAGAATAGAAACTACCCA
 |||||||||||||||||||||||||||||||||||
TACTGTTTTTATTCTATCACTTTTTGTTGCTCTGGGAGTCTTCAGAATAGAAACTACCCA
Supplementary Figure S1: Sequencing results of the BRCA1 mutated clones. Sequence of the genomic rearrangements of the clones was identified by amplicon-Seq of PCR products with two independent primer pairs flanking the Cas9 target sites.
Clone 14.3: 7 bp deletion - c.4511del7 / p.1505fs40* (171.7 kDa)
CCCTTCTAAATGCCCATCATTAGATGAT↓AGGTGGTACATGCACAGTTGCTCTGGGAGTCT
||||||||||||||||||||| ||||||||||||||||||||||||||||||||
CCCTTCTAAATGCCCATCATT-------↓AGGTGGTACATGCACAGTTGCTCTGGGAGTCT
 C P S L G G T C T V A L G V
FRIETTHLKRSSLRLLMWRSNSWKSLGHTIstop

## Slide 2
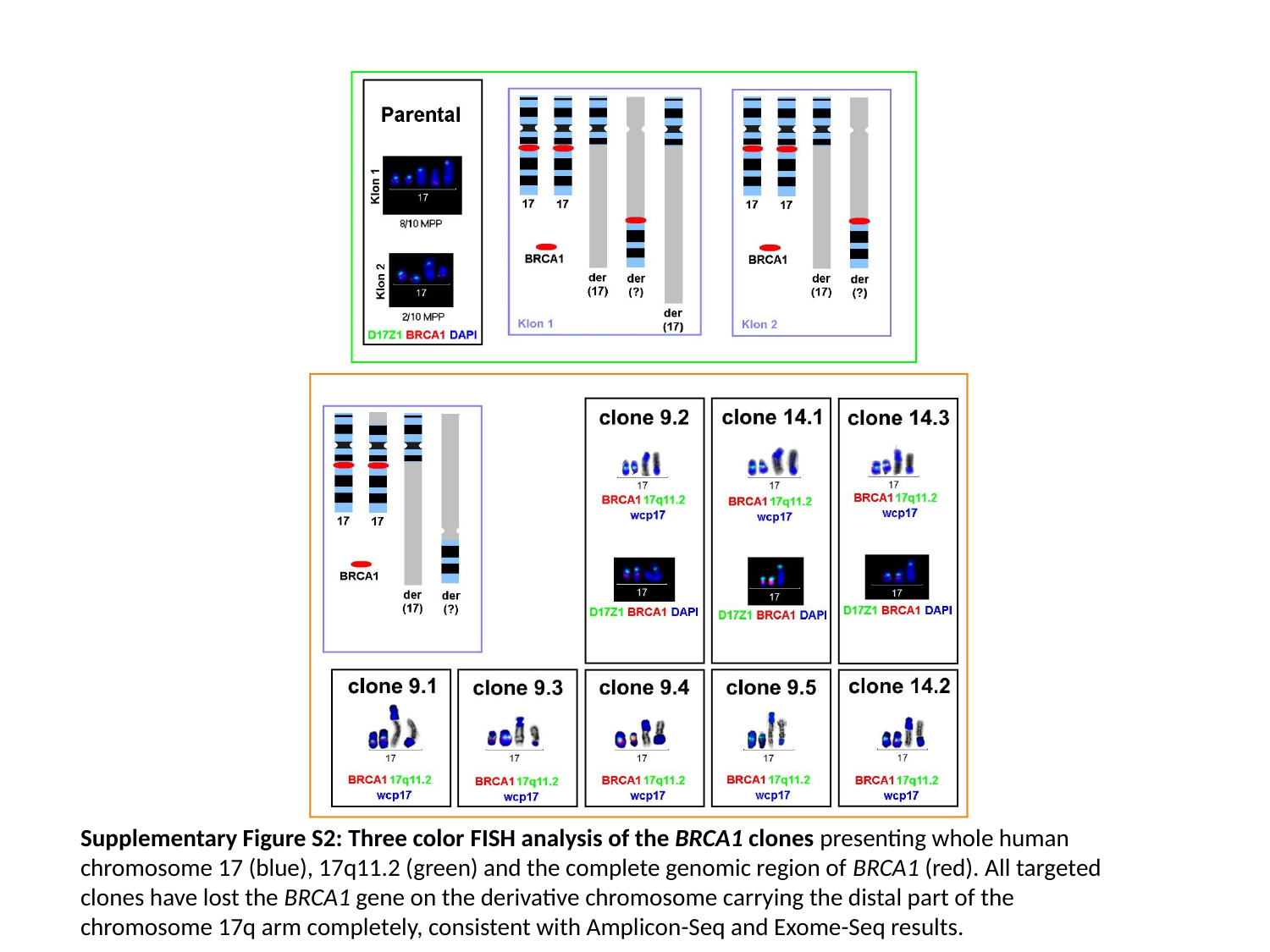

Supplementary Figure S2: Three color FISH analysis of the BRCA1 clones presenting whole human chromosome 17 (blue), 17q11.2 (green) and the complete genomic region of BRCA1 (red). All targeted clones have lost the BRCA1 gene on the derivative chromosome carrying the distal part of the chromosome 17q arm completely, consistent with Amplicon-Seq and Exome-Seq results.

## Slide 3
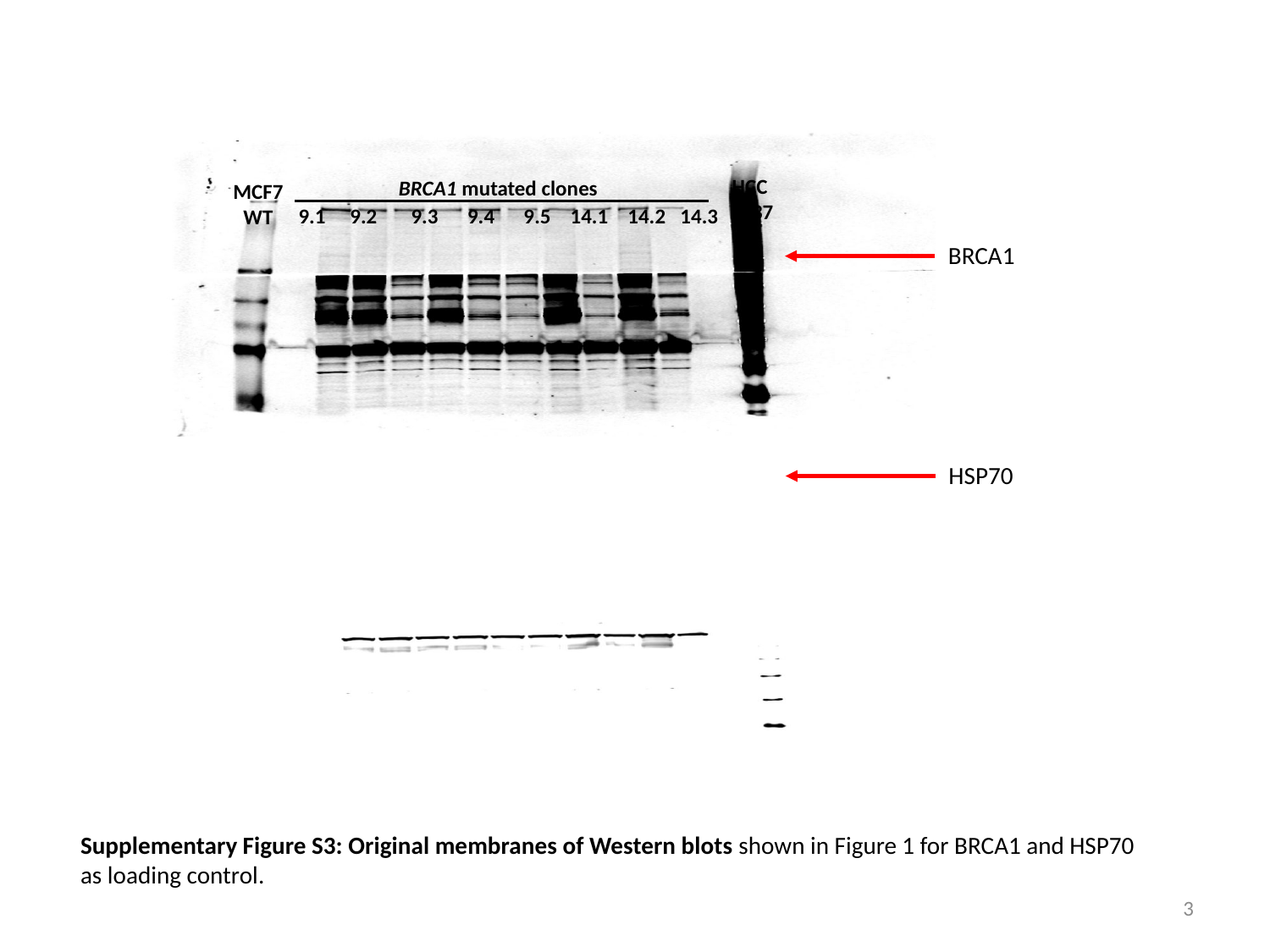

BRCA1
HCC
1937
BRCA1 mutated clones
MCF7
WT
9.1 9.2 9.3 9.4 9.5 14.1 14.2 14.3
HSP70
Supplementary Figure S3: Original membranes of Western blots shown in Figure 1 for BRCA1 and HSP70 as loading control.
3

## Slide 4
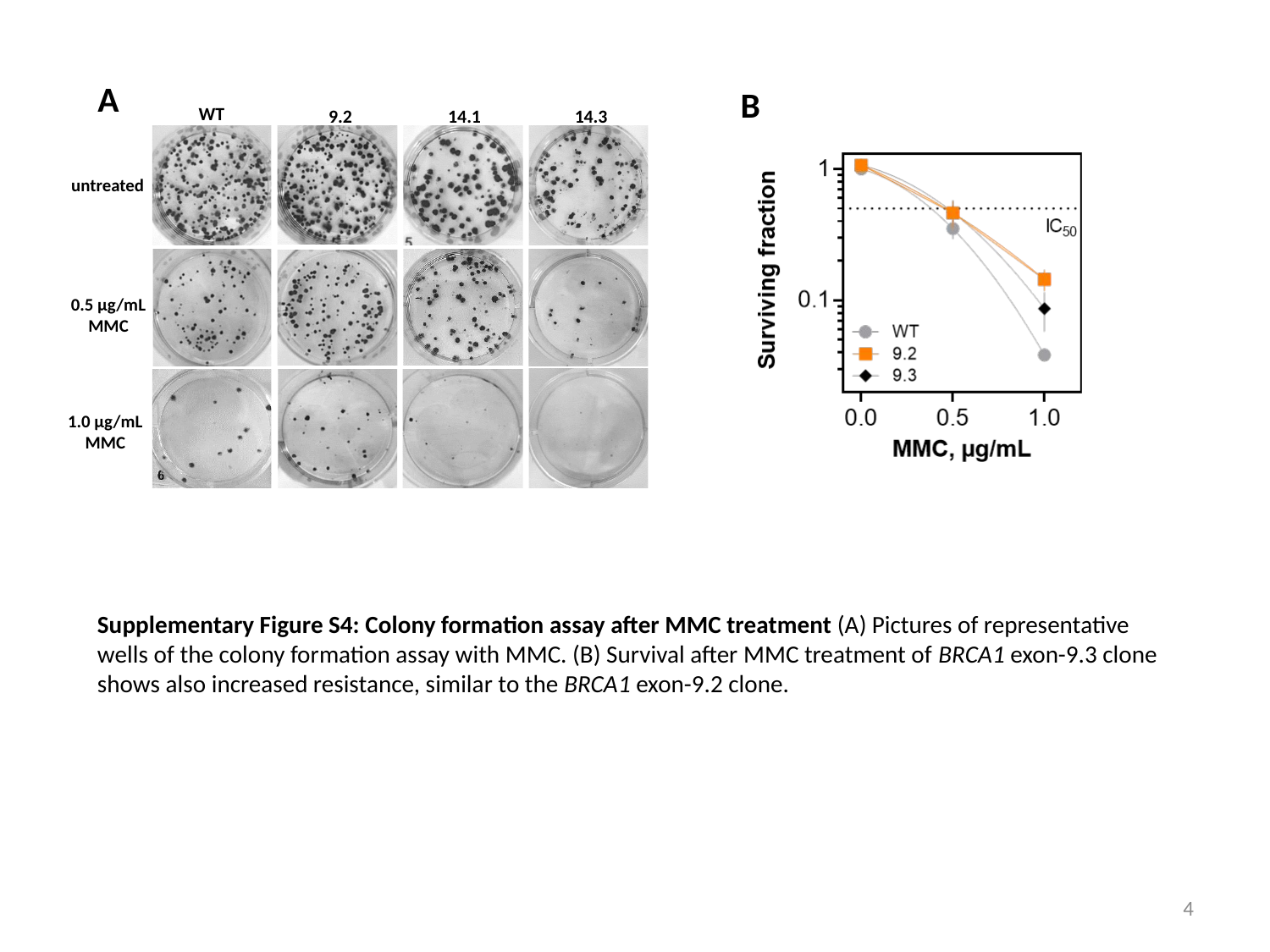

A
B
WT
14.3
9.2
14.1
untreated
0.5 µg/mL
MMC
1.0 µg/mL
MMC
Supplementary Figure S4: Colony formation assay after MMC treatment (A) Pictures of representative wells of the colony formation assay with MMC. (B) Survival after MMC treatment of BRCA1 exon-9.3 clone shows also increased resistance, similar to the BRCA1 exon-9.2 clone.
4

## Slide 5
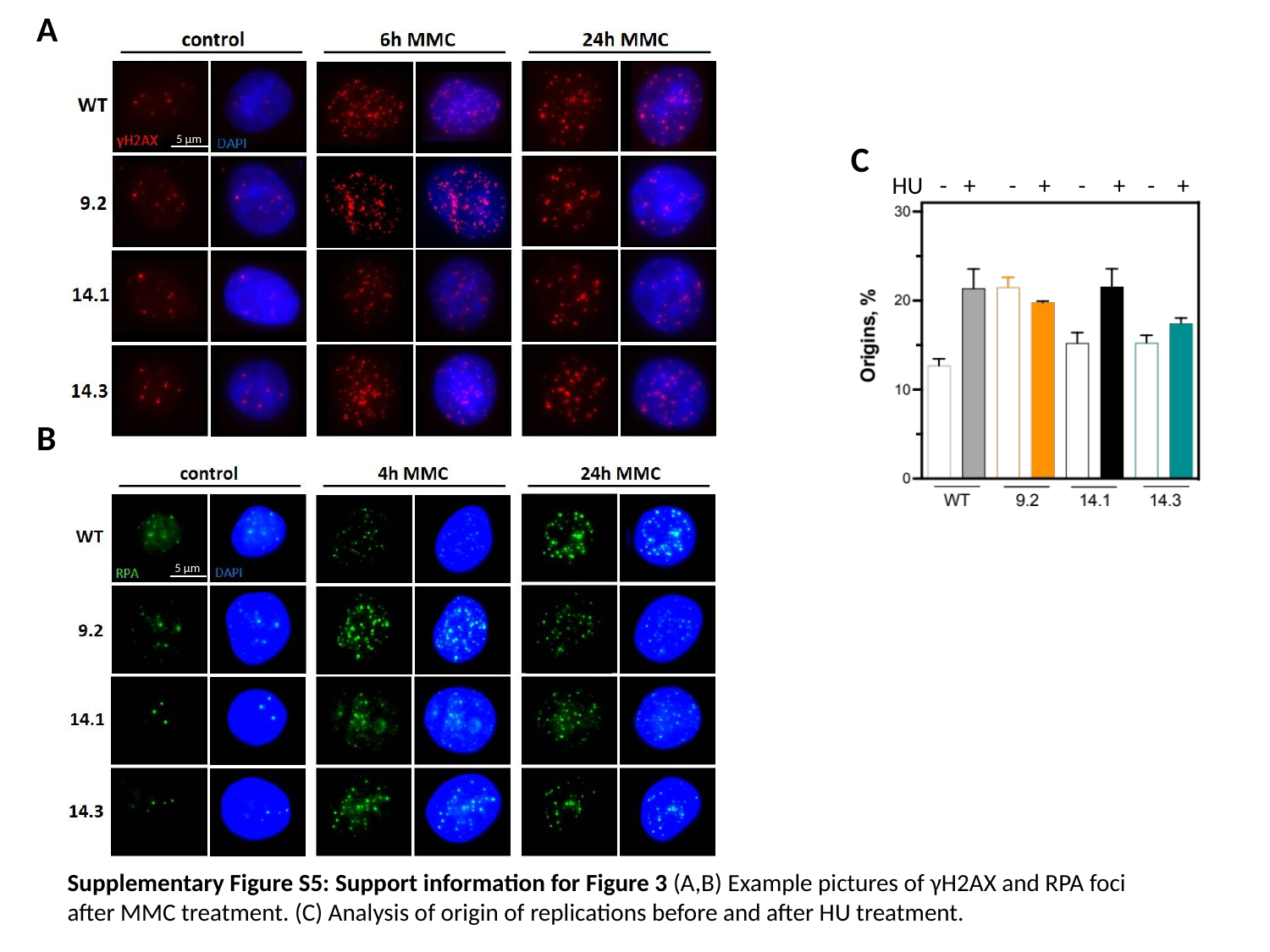

A
5 µm
C
HU - + - + - + - +
B
5 µm
Supplementary Figure S5: Support information for Figure 3 (A,B) Example pictures of γH2AX and RPA foci after MMC treatment. (C) Analysis of origin of replications before and after HU treatment.

## Slide 6
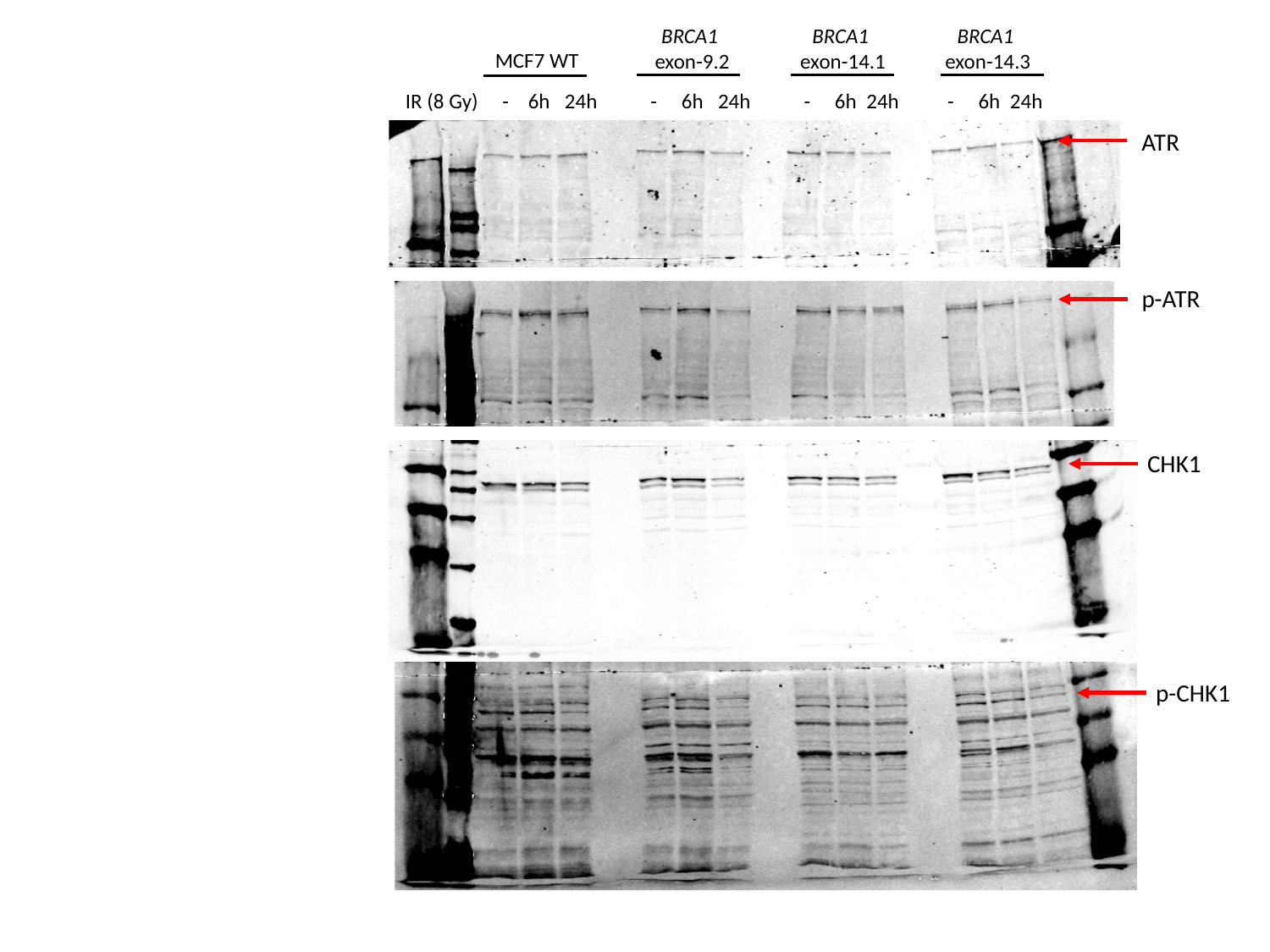

BRCA1
exon-9.2
BRCA1
exon-14.1
BRCA1
exon-14.3
MCF7 WT
IR (8 Gy) - 6h 24h - 6h 24h - 6h 24h - 6h 24h
ATR
p-ATR
CHK1
p-CHK1

## Slide 7
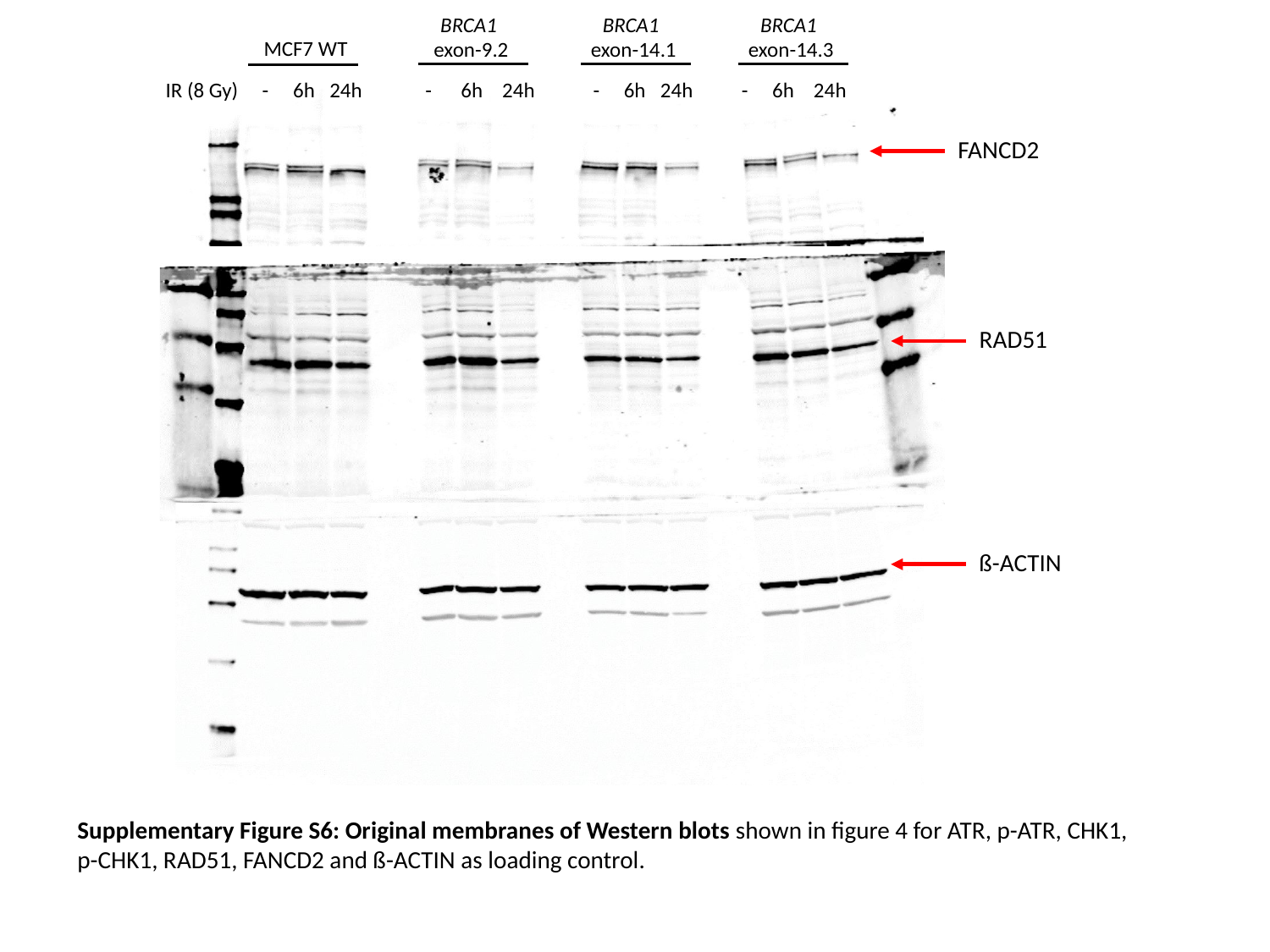

BRCA1
exon-9.2
BRCA1
exon-14.1
BRCA1
exon-14.3
MCF7 WT
IR (8 Gy) - 6h 24h - 6h 24h - 6h 24h - 6h 24h
FANCD2
RAD51
ß-ACTIN
Supplementary Figure S6: Original membranes of Western blots shown in figure 4 for ATR, p-ATR, CHK1, p-CHK1, RAD51, FANCD2 and ß-ACTIN as loading control.

## Slide 8
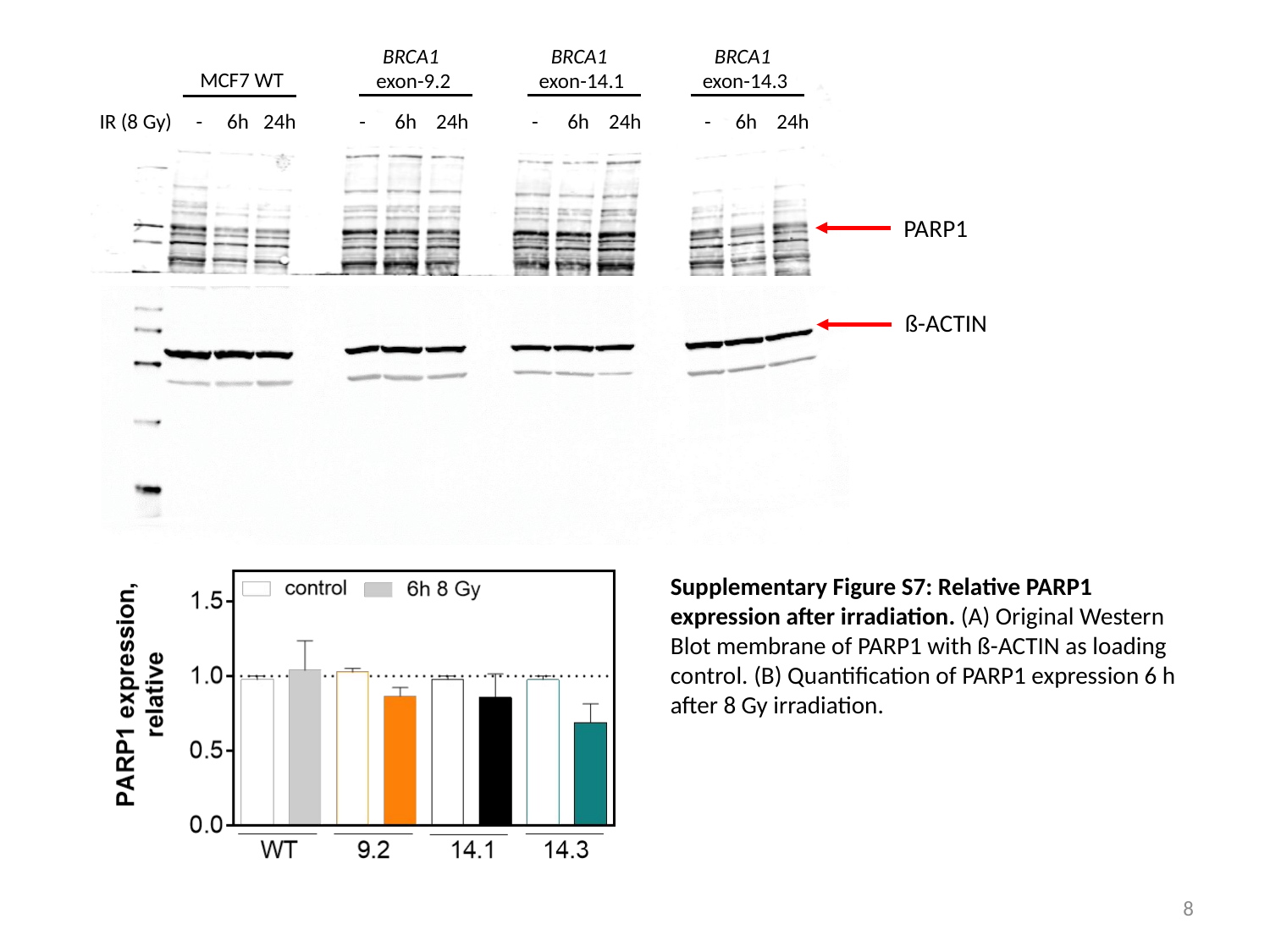

BRCA1
exon-9.2
BRCA1
exon-14.1
BRCA1
exon-14.3
MCF7 WT
IR (8 Gy) - 6h 24h - 6h 24h - 6h 24h - 6h 24h
PARP1
ß-ACTIN
Supplementary Figure S7: Relative PARP1 expression after irradiation. (A) Original Western Blot membrane of PARP1 with ß-ACTIN as loading control. (B) Quantification of PARP1 expression 6 h after 8 Gy irradiation.
8
